# Empathy Quotient and Systemizing Quotient in Elementary School Children with and without Attention-Deficit/Hyperactivity Disorder: A Comparative Study

**DOI:** 10.3390/ijerph18179231

**Published:** 2021-09-01

**Authors:** Agnes Lasmono, Raden Irawati Ismail, Fransiska Kaligis, Kusuma Minayati, Tjhin Wiguna

**Affiliations:** 1 Department of Psychiatry, Faculty of Medicine, Dr. Cipto Mangunkusumo General Hospital, Universitas Indonesia, Jakarta 10430, Indonesia; agnes.lasmono@ui.ac.id; 2 Child and Adolescent Psychiatry Division, Department of Psychiatry, Faculty of Medicine, Dr. Cipto Mangunkusumo General Hospital, Universitas Indonesia, Jakarta 10430, Indonesia; raden.irawati@ui.ac.id (R.I.I.); fransiska.kaligis@ui.ac.id (F.K.); kusuma.minayati@ui.ac.id (K.M.)

**Keywords:** empathy quotient, systemizing quotient, children, ADHD, EQ, SQ, Indonesia

## Abstract

This study compares the Empathy Quotient (EQ) and Systemizing Quotient (SQ) scores of elementary school children with and without ADHD. The study also examined their brain types and, because sex plays a big role in empathy and systemizing ability, compared the results of the boys and girls. This cross-sectional study involved 122 participants, including 61 parents of children with ADHD and 61 parents of children without ADHD. The EQ, SQ and brain types were obtained using the Empathy and Systemizing Quotient in children (EQ-/SQ-C), validated in the Indonesian language. Data was analyzed using the SPSS program version 20 for Windows, with a *p*-value < 0.05 for statistical significance. There was a significant difference in EQ between children with and without ADHD, the score being lower in children with ADHD. There was also a significant difference in SQ among girls with and without ADHD, but not in boys. The brain types in both groups were not significantly different. The results indicate that children with ADHD have a lower ability to empathize compared to children without ADHD. Systemizing abilities were significantly lower in girls with ADHD than in girls without. Therefore, an intervention program focusing on improving empathy and systemizing ability needs to be developed in the community.

## 1. Introduction

Attention-deficit/hyperactivity disorder (ADHD) is a neurodevelopmental disorder that has the main characteristics of persistent failure in sustaining attention and/or hyperactive or impulsive behavior. These characteristics are significant in at least two situations and have an onset before the age of 12 years old [1]. ADHD can be found worldwide, with a prevalence of 5 to 8% in school-age children [2]. The presence of ADHD in children is associated with difficulties in social relationships and academic achievement. They may experience rejection from peer groups, have limited friends, be less popular, or fail to advance in class. These difficulties may contribute to wider and longer-term impacts on their lives, which are also related to poor self-image as a consequence. Since ADHD is quite common and is one of the most common conditions in psychiatric outpatient clinics, these difficulties in social functioning and academic areas need to be considered as a target of therapy [3,4,5].

The social interaction is an important milieu for children especially for their empathy and psychosocial development. During the preschool age, children tend to think egocentrically and may not see things from others’ perspectives. However, as language and cognition develop, they gradually learn to express more complex emotions (such as guilt, envy, etc.), share things, and try to understand about other people. At the end of preschool age, children have the capacity for empathy and learn that others may think and feel differently from them. These abilities help them through their interaction with others during school age to adulthood [6]. Peer relationships in children begin especially at the beginning of school age. According to Erik Erikson, school age (6–13 years old) is the main stage that determines social functioning in children. They will learn to engage in activities beside and with other people. In a study conducted by Nathania, Mahdiyyah, Chaidir, Phalapi and Wiguna [7], there was a significant relationship between empathy and peer-relationship in elementary school children, in which empathy contributes to better social function in children. By definition, empathy is the ability to identify and predict the mental state of others, and to respond with the appropriate emotion. Empathy is developed from the moment the child is born. McDonalds and Messinger [8] stated that newborns already begin to show signs of empathy, known as reflective crying. Newborns who listen to other babies crying often show a suffering reaction by crying. After that, the development of empathy continues with other suffering expressions in infants, empathic responses and helping behavior in toddlers, cognitive empathy in preschool age children, and empathy as a stable trait for young adults [6,7,8].

Empathy, according to Goldenfeld, Baron-Cohen and Wheelwright [9], is reciprocal and competitive with one’s systemizing ability. Someone with more empathy tends to have lower systemizing ability, and vice versa. Systemizing is the ability to form or analyze systems according to the rules of the physical environment and to predict a systems’ behavior according to the rules. The systems in human behavior and cognition include technical, nature, abstract, motoric, social and other systems that can be arranged. Systemizing will help children learn about definite and lawful phenomena; hence, it may contribute to better performance in subjects such as mathematics, science, technical or other rule-based subjects for children. On the other hand, during empathizing, someone will not expect a lawful relationship between others’ emotions and behavior. Even so, empathy can help children in their social lives [9,10].

Baron-Cohen, Richler, Bisarya, Gurunathan and Wheelwright [10] developed the Empathy Quotient (EQ) and the Systemizing Quotient (SQ) as instruments to assess the empathy and systemizing ability in adults. Auyeung, Wheelwright, Allison, Atkinson, Samarawickrema and Baron-Cohen [11] modified the instrument to expand its use to children. With the EQ/SQ score acquired from the instrument, Goldenfeld et al. [9] introduced a formula which uses the EQ and SQ score of the identified person and compares it to the typical population’s EQ and SQ score. The results may explain the more dominant drive or cognitive style of a person, which is called the “brain type”. There are five “brain types”, which are extreme empathy, empathy, balanced, systemizing and extreme systemizing [9,10,11].

Both empathy and systemizing abilities are relatively stable in life. Thus, individual differences in these cognitive styles should have been observed since childhood [10,11]. So far, the EQ/SQ study in ADHD has only been performed in adult patients, and the results vary. Aviles et al., 2014 found SQ in adults with ADHD is lower compared to adults without ADHD, while Groen, den Heijer, Fuermaier, Althaus and Tucha (2018) found lower empathy and a more systemizing cognitive style in adults with subclinical ADHD [12,13]. Therefore, this study aimed to identify the EQ and SQ, as well as the brain type of children with ADHD, and compare the results to children without ADHD. The purpose was to produce basic data on differences in EQ and SQ, as well as brain type in children with and without ADHD, which can be used as preliminary data for further research in the field of child and adolescent mental health. The results of this study can also be used as a reference in developing mental health service programs, especially to improve empathy in children with ADHD. By knowing the empathy and systemizing ability of children with ADHD starting from the time they are in elementary school, this ability can be developed earlier, so that psychosocial impacts that may not be good, such as poor learning achievement, problems in self-image, behavioral disorders (such as delinquency or the commission of criminal acts), problems in peer relationships, and conflicts in the family may be reduced. Because sex plays a big role in empathy and systemizing ability, this study examined the results specified for both boys and girls.

## 2. Materials and Methods

### 2.1. Participants

This was a cross-sectional study. A total of 122 elementary school-aged children and their parents participated in this study. They were categorized into two groups: with ADHD group (61 pairs) and without ADHD group (61 pairs). Children with ADHD were recruited from Child and Adolescent Psychiatry outpatient clinic at dr. Cipto Mangunkusumo General Hospital, Central Jakarta. Meanwhile, children without ADHD were selected from one public primary school at Central Jakarta. All research subjects joined the study consecutively based on parents and children willingness to participate into the study. The inclusion criteria for children without ADHD were those with Conners’ Abbreviated Teacher/Parent Rating Scale (CATPRS) score of less than 12 and never been diagnosed as ADHD based on parent report. Furthermore, the inclusion criteria for children in the ADHD group were children with diagnosed ADHD by a child and adolescent psychiatrist from the Dr. Cipto Mangunkusumo General Hospital, Child and Adolescent Psychiatry outpatient clinic. All parents had at least a junior high school education and agreed to participate in this study by signing informed consent. The diagnosis of ADHD was based on the International Classification of Disease-10 (ICD-10) diagnostic criteria for ADHD. Furthermore, children with ADHD have been medicated by using methylphenidate hydrochloride immediate release for several months with dose range 5–20 mg per day in divided dose. The exclusion criteria for this study were children with severe psychiatric disorder (including autistic disorder, moderate to severe mental retardation, psychotic disorder, major depressive disorder and bipolar disorder) and physical disability. The statistical power for this study was set 80% and *p*-value < 0.05 to determined the statistical significancy. The number of participants included in this study was accordant to the sample size calculation for comparison study. The Ethics Committee of the Faculty of Medicine Universitas Indonesia has approved the study protocol (KET-387/UN2.F1/ETIK/PPM.00.02/2020 on 6 April 2020).

### 2.2. Instruments

The socio-demographic data of both parents and children were collected using a questionnaire that was specifically developed for this study. Conners’ Abbreviated Teacher/Parent Rating Scale (CATPRS), which is an abbreviated version from Conners’ Teacher Rating Scale and Conners’ Parent Rating Scale with 10-statements related to children’ behavior, was also used to screen out the children with ADHD from the elementary school. The rating of the instruments was on a Likert scale, with scores ranging from zero (not at all) to three (almost always) for each statement. The instrument was valid and reliable for use, with a cut-off score of 12 [14]. The MINI-Kid instrument was also used as a structured diagnostic interview for screening children with severe mental disorders, including psychotic disorders, depression, and bipolar disorder. For children under 13 years of age, the interview was conducted with the children being accompanied by parents. An instrument that has been translated into Indonesian was used. The instrument has varied sensitivity and specificity to each psychopathology, ranging from 61 to 100% for sensitivity and 81 to 100% for specificity [15,16].

The Empathy and Systemizing Quotient for Children (EQ- & SQ-C), which is a parent-rating scale to assess empathy and systemizing quotients, was also used in this study. The Indonesian version of the EQ- & SQ-C was assessed for reliability with an internal consistency of Cronbach’s alpha 0.957 for EQ-C, 0.962 for SQ-C, and 0.979 for overall EQ- & SQ-C. Each point of this instrument also has good construct and content validity. The Indonesian version of this questionnaire has 38 items in total, with 18 statements for EQ-C and 20 statements for SQ-C. Parents have four different choices for each statement: “strongly agree,” “slightly agree,” “slightly disagree,” and “strongly disagree.” For the empathy quotient, statements 9, 11, 15, 18, 19, 28, 29, and 31 will be given a score of 2 points if “strongly agree” is chosen and 1 point if “slightly agree” is chosen. For statements 1, 3, 4, 6, 12, 20, 23, 26, 37, and 38, the 2 points are given if the parents choose “strongly disagree” and 1 point for “slightly disagree.” For systemizing quotients, the scoring for statements 5, 7, 8, 13, 17, 21, 22, 24, 25, 27, 30, 32, 34, and 35 is 2 points for those who answered “strongly agree” and 1 point for “slightly agree.” For statement numbers 2, 10, 14, 16, 33, and 36, the 2 points are given for a “strongly disagree” answer and 1 point for “slightly disagree.” The rest of the choices were given a score of 0 points. The maximum total points were 36 for EQ and 40 for SQ. If there is no answer in a minimum of five statements, then the result will be considered invalid, and the data will not be analyzed [7,17].

After the EQ and SQ scores were calculated, the D score (difference between standardized EQ and SQ) and C score (combined score of standardized EQ and SQ) were calculated. The formulas used were:

D = (standardized SQ − standardized EQ)/2

C = (standardized SQ + standardized EQ)/2

Standardized EQ or SQ is obtained by subtracting mean EQ or SQ of typical population from observed EQ or SQ score, then divide it with the possible maximum score of EQ or SQ. The D score indicates the differences between empathy and systemizing ability, which can be used to determine the brain types of children. The brain types were extreme empathy (with percentile of D score < 2.5), empathy (percentile of D score between 2.5 to 35), balance (percentile of D score between 35 to 65), systemizing (percentile of D score between 65 to 97.5), and extreme systemizing (percentile of D score > 97.5). The C score indicates the accumulation between empathy and systemizing ability, which can be used to see whether the two abilities are reciprocal to each other or not.

### 2.3. Data Analysis

All of the data were analyzed using SPSS version 20.0. Descriptive data for socio-demographics and brain types according to EQ and SQ are shown in proportion. EQ and SQ scores are shown in the form of mean and standard deviation if the data were distributed normally, and median and range if not normally distributed. The relationship between ADHD and brain type was analyzed with chi-square or alternative Kolmogorov-Smirnov tests. Furthermore, the mean difference of EQ and SQ, and the D and C scores between groups was analyzed using an independent t-test for normally distributed data, and the Mann-Whitney U test if not.

## 3. Results

Between May 2020 and February 2021, 122 participants completed the questionnaire. The participants’ characteristics are listed below. Table 1 describes the characteristics of the parents, while Table 2 describes the characteristics of the children.

**Figure 1:**
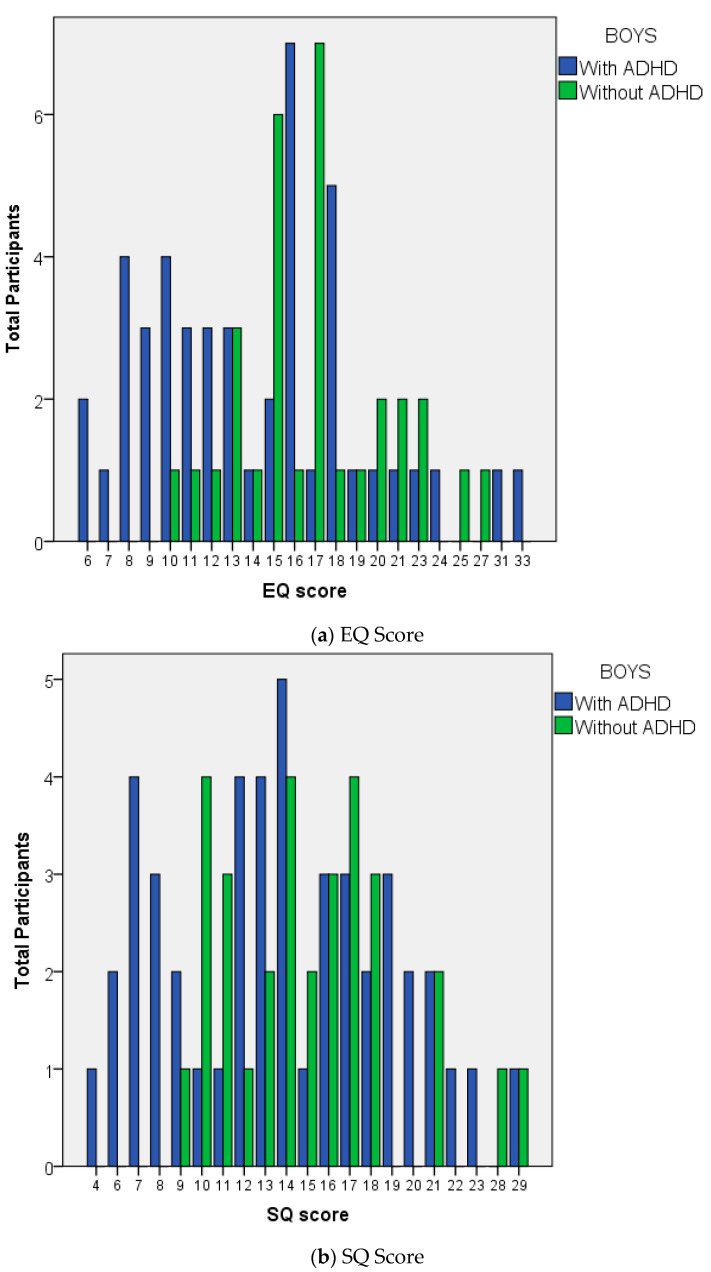
Distribution of (**a**) EQ score and (**b**) SQ score; in boys with ADHD (in blue) and boys without ADHD (in green). Lower EQ scores were found in boys with ADHD group.

With regard to the mean difference of the EQ scores between both groups, the data in the boy subgroup was not distributed normally; thus, this study used the Mann-Whitney U test for analyzing the EQ scores of the boys. However, the other data (EQ score in girls and SQ score in both subgroups) were distributed normally and were analyzed using an independent t-test. As some of the data were not distributed normally, this study used the mean, standard deviation (SD), median, and range (minimum-maximum) for descriptive purposes. The results are presented in Table 3. There was a significant difference between both groups with regard to EQ scores (*p* = 0.010 in boys and *p* = 0.002 in girls), and a statistically significant difference in SQ scores between both groups (with and without ADHD) in the girls subgroup (*p* = 0.008). However, the SQ scores in the boy subgroup did not differ significantly (*p* = 0.288). The effect of ADHD that may influence EQ and SQ scores in this study was small to large difference effect size (ranged from 0.29 to 1.29) (Table 3, Figure 1).

**Figure 2:**
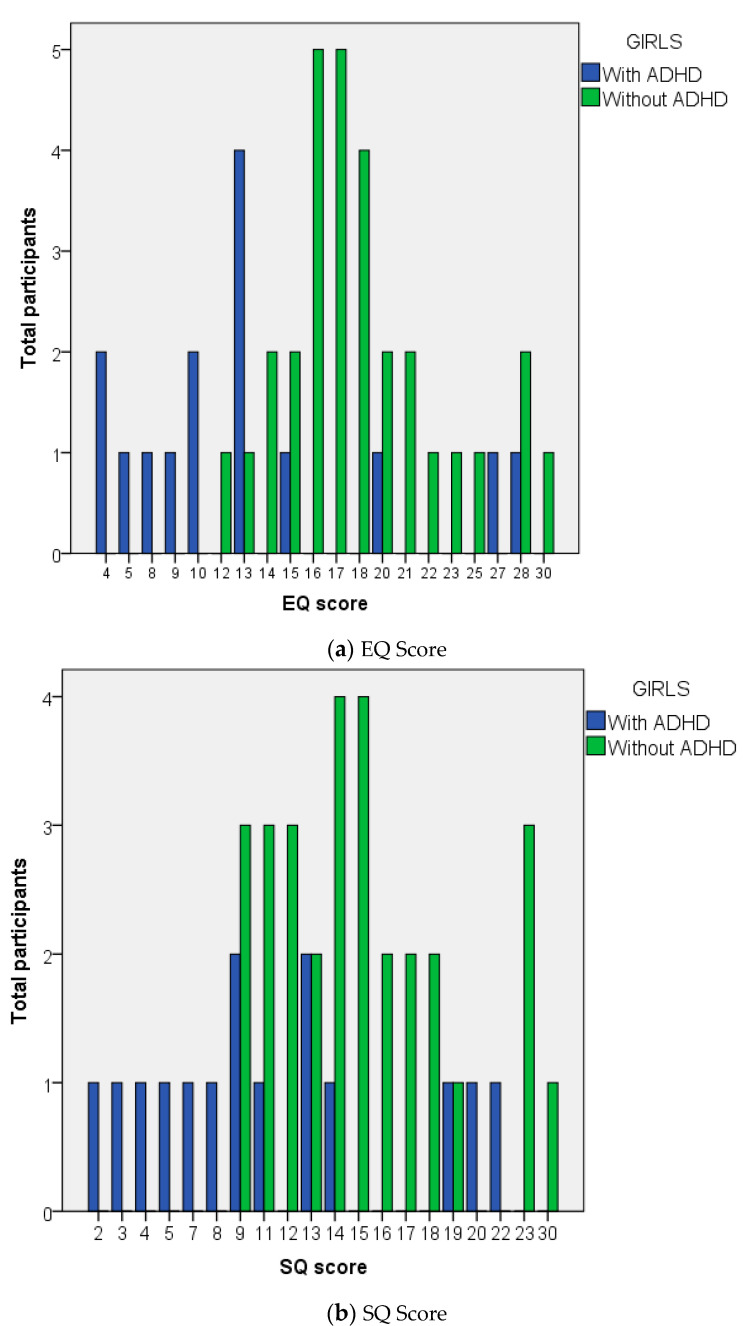
Distribution of (**a**) EQ score and (**b**) SQ score; in girls with ADHD (in blue) and boys without ADHD (in green). Lower EQ scores were found in girls with ADHD group.

The mean (SD) of the EQ scores in the without ADHD group, regardless of the sex-differences, is 17.80 (4.29), while the mean (SD) of the SQ scores is 15.30 (4.72) (Figure 2). Using these scores, this study counted the standardized and standardized SQ scores to determine the D score, and then classified the participants into five different brain types. The D score with percentile < 2.5, with a score of D < −0.16 identifies participants as part of the extreme empathy group. D score with percentile 2.5. to 35 with the score of D −0.16 to <−0.016 identifies participants as part of the empathy group, D score with percentile 35 up to 65 with the score of D −0.016 to <0.029 identifies participants as part of the balance group, D score with percentile 65 up to 97.5, with a score of D 0.029 to <0.105 identifies participants as part of the systemizing group, and D score with percentile ≥ 97.5 with a score of D ≥ 0.105 identifies participants as part of the extreme systemizing group. The distribution of brain types in the groups with and without ADHD is listed in Table 4. For the analysis, this study could not conduct the chi-square test because the criteria for chi-square were not fulfilled (40% cells had an expected count less than 5). Because of this, the study used the Kolmogorov-Smirnov test and found no significant difference between the groups (*p* = 0.385).

**Figure 3:**
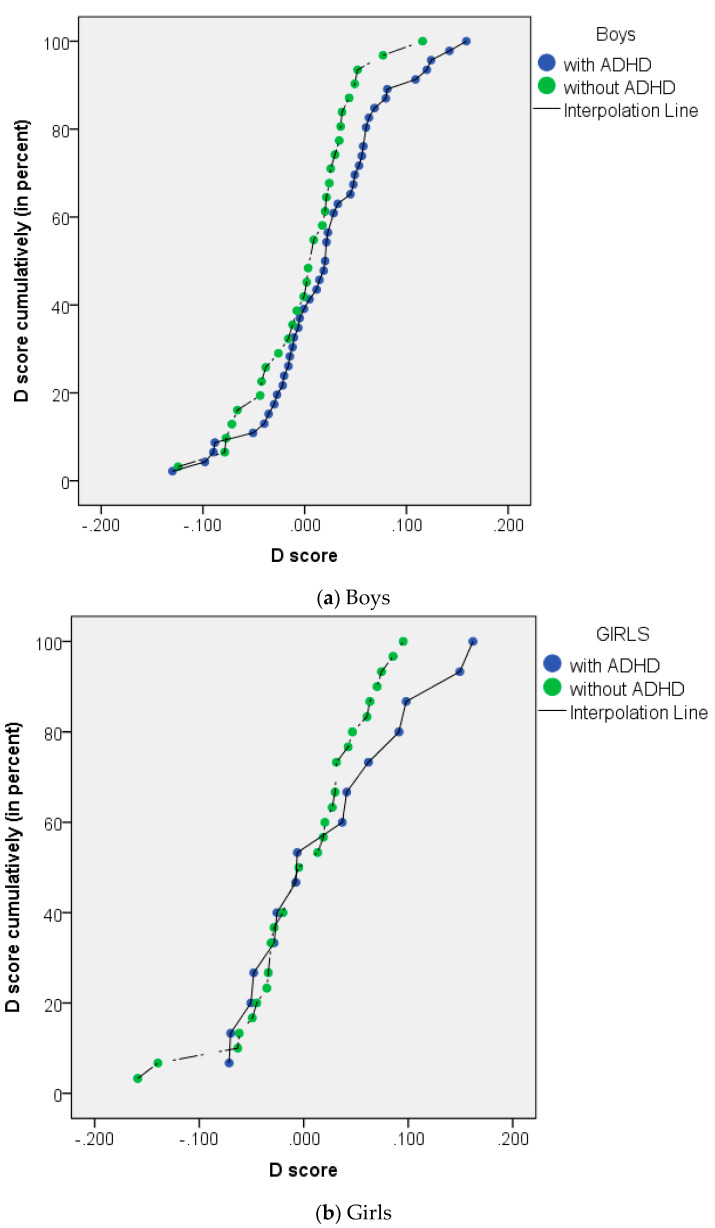
Distribution of D scores in (**a**) boys and (**b**) girls with ADHD (in blue) and without ADHD (in green). No significant difference found between with ADHD and without ADHD groups in both sexes.

For the boys, the mean (SD) D scores of the ADHD group and the without ADHD group were 0.019 (0.063) and 0.000 (0.052), respectively. For the girls, the mean (SD) D scores of the ADHD group and the without ADHD group were 0.022 (0.077) and 0.000 (0.064), respectively. Figure 3 shows the distribution of the D score in boys and girls, with and without ADHD. An additional analysis of the mean difference in D scores between the groups with and without ADHD was conducted using an independent t-test, and no statistically significant difference between the groups was found (*p* = 0.184 in boys and *p* = 0.334 in girls).

**Figure 4:**
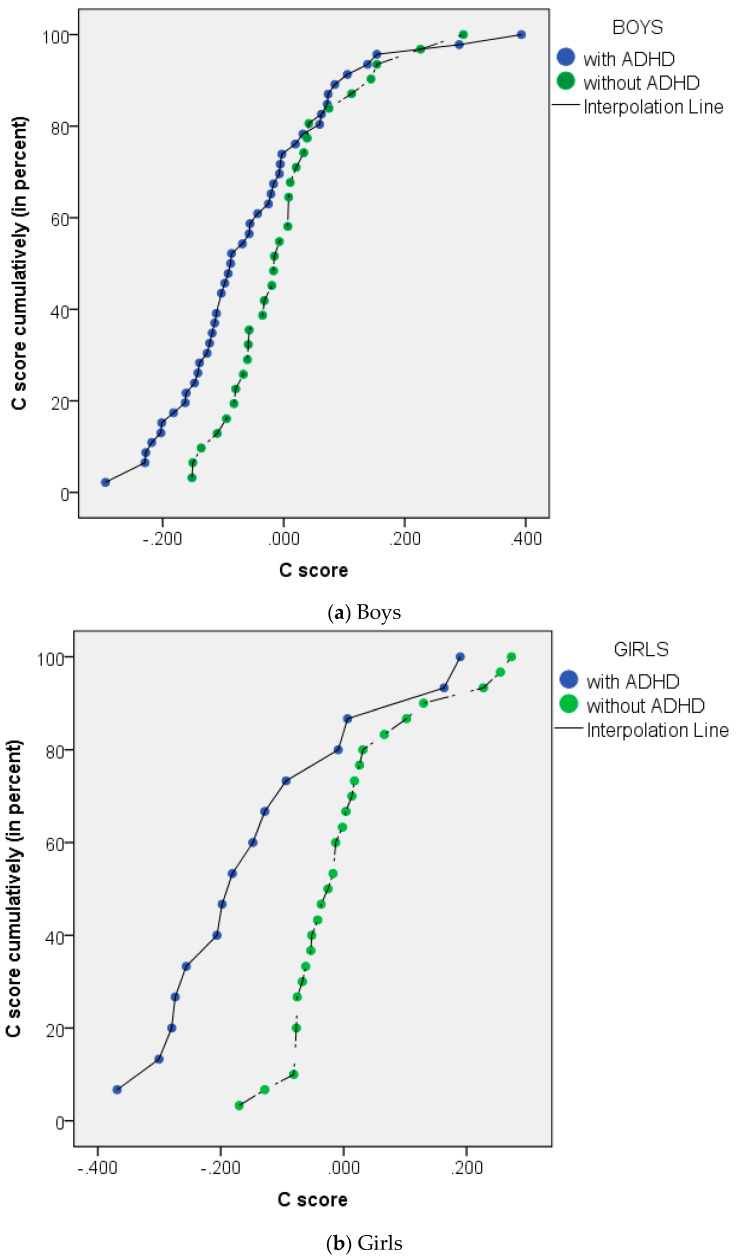
Distribution of C scores in (**a**) boys and (**b**) girls with ADHD (in blue) and without ADHD (in green). No significant difference found between boys with ADHD and without ADHD, but there were significant differences between girls with ADHD and without ADHD.

The mean (SD) C score was also calculated. For the boys, the mean (SD) of the C score of the ADHD group was −0.050 (0.137) and the group without ADHD’s was 0.000 (0.106). For the girls, the mean (SD) of the C score of the group with ADHD was −0.139 (0.165), and the group without ADHD’s was 0.009 (0.110). The C score distribution shown in Figure 4 shows differences between the groups with and without ADHD. In the ADHD group, the accumulation of the standardized EQ and the standardized SQ (indicated by the C score) was lower, but when analyzed statistically, there was no statistically significant difference with regard to the boys (*p* = 0.099). However, with regard to the girls, the results were significantly different (*p* = 0.001).

## 4. Discussions

In this study, the Empathy Quotient and the Systemizing Quotient were determined using the EQ- & SQ-C questionnaire that was completed by 122 parents of elementary school children. To our knowledge, this is the first study to compare the EQ, SQ, and brain types according to EQ and SQ in children with and without ADHD. This study reports the results in boys and girls subgroup, taking into account the role of gender in empathy and systemizing ability. Some studies have found that boys have lower empathy than girls. Chaidir et al., 2019 observed that boys tend to have a more systemizing brain type, while girls tend to have more empathizing brain type [18]. Auyeung et al., 2009 also stated that there is a boy and girl difference with regard to brain type that may be influenced by children’s biological condition [11,19]. Moreover, other factors such as children’s hobbies or social skills may also affect the difference in brain types between boys and girls [20]. ADHD is also more prevalent among boys [21]. Therefore, this study differentiates the analysis of the sex of the children (boys or girls).

The results of the EQ in boys with and without ADHD were significantly different (*p* = 0.010). A significant difference was also observed in girls (*p* = 0.002). In both boys and girls, a lower EQ score was found in the ADHD group. The results indicate that in children with ADHD, regardless of gender, there is a difference in empathy. Lower empathy means that children with ADHD have difficulties in identifying others’ mental states and responding appropriately. These results are concordant with the study conducted by Groen et al. [13] who studied empathy in adults with subclinical ADHD. Groen et al., 2018 concluded that there was decreased emotional empathy in the group with subclinical ADHD compared to the group of adults without ADHD [13]. The lower EQ score in the ADHD group may be influenced by difficulties in maintaining attention, hyperactivity, and impulsive behavior that are related to their ability to recognize cues from others and to listen to others [22].

Other studies on empathy in children with ADHD show mixed results. Parke, Becker, Graves, Baily, Paul, Freeman and Allen [23] found that children with ADHD have worse scores compared to the controls with regard to emotional recognition, pragmatic language, cognitive theory of mind, and cognitive empathy. Cognitive theory of mind refers to children’s ability to understand other people’s thoughts, while cognitive empathy refers to the ability to see the perspective of others [23]. However, Marton, Wiener, Rogers, Moore and Tannock (2009) found no difference between children with ADHD and the controls with regard to self-reported empathy, while in the parent-report questionnaire, empathy was lower in children with ADHD [24].

Empathy consists of two components, i.e., cognitive and affective. The cognitive component of empathy may be affected by deficits in executive functioning [23]. According to Suryani (2012), impaired executive function, especially in working memory, inhibition, and organization of the material domain, was found in 49.5% of elementary school children with ADHD in DKI Jakarta [25]. Deficits in executive functioning in children with ADHD may cause said children to face difficulties when relationship transactions become more intense. They may find it difficult to understand cues from others, and if the ability to recognize others’ emotional cues is difficult, then recognizing how they should appropriately respond may also be difficult [22,23,26].

As for the SQ, the results for boys did not differ significantly between the groups (*p* = 0.288), while for the girls, the difference was significant (*p* = 0.008). However, in both boys and girls, children with ADHD had lower SQ scores than those without ADHD. Consistent with our study, a study by Aviles et al. (2014) also showed that the SQ score in adults with ADHD was lower than that in adults without ADHD, with a mean score of 29.5. Aviles et al., 2014 used a version of the instrument with a maximum score of 80. [12] However, the questionnaire used in this study was in Indonesian language that had different total statements in SQ and a maximum score of 40. Regardless of the version, the result was parallel—the SQ score was lower in the ADHD group than in the non-ADHD group. A lower systemizing score mean that in children with ADHD, the ability to form, analyze, and predict the system is lower. This result may be influenced by the difficulties in obtaining details and lower executive functions that are needed for systemizing. Unlike individuals with autism spectrum disorder (ASD) who have high systemizing skills, individuals with ADHD lack the ability to focus on details that are important for systemizing. Moreover, lower executive function was also found in children with ADHD, which may be related to information processing and integration, as well as other skills that may help in completing special systemization tasks [12,13].

In the study by Aviles et al., 2014, a significant difference in SQ between sexes in ADHD was also found, in which boys scored higher than girls. This result may be related to the results of the present study, in which no significant difference was found in boys with ADHD, while a significant difference was found in girls with ADHD, compared to those without ADHD [12]. When considering the mean SQ in this study, the SQ in the group without ADHD for either boys or girls was higher than that in boys with ADHD, and the SQ in boys with ADHD was higher than that in girls with ADHD. For the boys, the difference between the groups with and without ADHD is not significant and may be caused by mental rotation ability—an important aspect of systemizing—that was still high. Some statements in the SQ instrument related to the capacity for visualization of spatial relations. Linn and Petersen [27], and also Mackintosh and Bennett [28], found superiority in boys on mental rotation tasks, compared to girls. The differences in the SQ between sexes may be related to this mental rotation ability [27,28,29].

In this study, there were no significant differences found in brain types based on EQ and SQ between children with and without ADHD (*p* = 0.385). This result may be influenced by the medication being administered to children with ADHD. Most of the participants in the ADHD group had previously received methylphenidate as a medication for their ADHD symptoms. In addition, the data were collected during the Covid-19 pandemic, when children were stayed mostly in their houses. Empathy and systemizing skills may not be rated accurately by parents because both skills are clearer when observed in the school situation, when they are faced with more friends, more complex interactions, and more tasks that may need their systemizing skills. Another factor that may be taken into consideration is that in this study, the questionnaire was filled out by the parents. Parents usually interact with children one-on-one. As stated by Deschamps, Schutter, Kenemans, and Matthys (2015), when interacting with a friend in a quiet environment, children with ADHD are not associated with decreased empathic responses to other people’s sadness and difficulties [30]. Even so, these results may be interpreted as the cognitive style, which may be related to the children’s choice of higher education level (science or humanities), being the same between children with and without ADHD [31].

The D score was also analyzed in this study to examine whether there is a tendency towards a systemizing brain type, as Groen et al. [13] found in the ADHD group. The D score represents the individual differences in cognitive style and is not related to an individual’s IQ or age [32]. Groen et al. [13] stated that a positive score of D is an indication of a systemizing or extreme systemizing brain type, while a negative score indicates an empathy or extreme empathy brain type. A D score close to zero indicates a balanced brain type. This study found that in the ADHD group, the D score tended to be positive compared to the group without ADHD, and in the group without ADHD, the D score was close to zero. Higher D scores may be caused by higher SQ or lower EQ, and vice versa. A lower D score may be caused by lower SQ, higher EQ, or little difference between both abilities. However, when analyzed statistically in this study, the positive D score in the ADHD group was not statistically different from that of the non-ADHD group, with *p* = 0.184 in boys and 0.334 in girls.

The C score in this study in the boys subgroup did not differ significantly (*p* = 0.099). However, for the girls, the results were significantly different (*p* = 0.001). The significant difference means that empathy and systemizing abilities are not in a reciprocal relationship with each other in the girls subgroup. Reduced empathy is not always followed by higher systemizing abilities, and vice versa; reduced systemizing ability is not always followed by higher empathy in girls with ADHD. Although some studies found that empathy is reciprocal with systemizing, Wakabayashi, Baron-Cohen, Wheelwright, Goldenfeld, Delaney, Fine, Smith, and Weil (2006) stated that SQ and EQ were almost unrelated to each other, with the correlation between EQ and SQ being close to zero [9,31]. Groen et al. (2018) also stated that ADHD differs from a typical ASD-like profile; in ASD, previous studies found that reduced empathy is accompanied by increased systemizing scores [13]. These results were concordant with the results of the present study in the girls subgroup. The SQ and EQ in girls with ADHD are considered not optimum—the combined score is lower than that of girls without ADHD. In boys, the difference was not significant, which may be caused by a higher SQ score, which is found in this study as showing no difference from the control.

Neuroanatomical factors may also play a role in the independency between EQ and SQ, as the brain part included is different. Lai, Lombardo, Chakrabarti, Ecker, Sadek, Wheelwright, Murphy, Suckling, Bullmore, MRC AIMS Consortium, and Baron-Cohen [32] used structural MRI imaging and looked at how neuroanatomical factors contributed to the differences between empathy and systemizing. The systemizing ability is associated with an increased volume of gray matter in the cingulate and dorsal medial prefrontal areas, while empathy is associated with the larger hypothalamus and ventral basal ganglia [32].

Due to the role of executive function in empathy and systemizing abilities, this study considered IQ, which may have influenced the results. However, in several studies that have been conducted, it was found that while systemizing was related to mental rotation, it was not related to intelligence [27,33]. The same applies to empathy, which was found to be unrelated to verbal IQ in the study by Chapman et al., 2006, although verbal IQ was found to be associated with more complex social words [20]. The D score was also found to be unrelated to IQ in some studies. In the study conducted by Lai et al. [32], it was found that the brain regions that were included in deductive reasoning were not associated with systemizing brain types. Lai et al., 2012 stated that the questionnaire tends to measure character traits and drives rather than intelligence [32]. In this study, children with mild intellectual disabilities were not excluded. Assessment of mild intellectual disability in the subject cannot be carried out because of the pandemic, which makes it impossible to invite children to the outpatient clinic for IQ examination. However, the participants in the ADHD group included in this study mostly had no intellectual disabilities, and even if they had, they were still at a mild level that could be seen by their ability to carry out their daily functioning activities independently. This study has two limitations that may possibly associate with the results. First, this study did not exclude children with intellectual disabilities. Secondly, ADHD children in this study had received medication for prior the study that may also influence the EQ and SQ capacity.

## 5. Conclusions

This study found significant differences in EQ (in both boys and girls) and SQ (in girls); however, there was no significant difference in brain types between elementary school children with and without ADHD. Further studies are needed that included more factors that may influence EQ and SQ in children with ADHD. Thus, better understandings of the brain types in this population, according to EQ and SQ can be generated. This study also suggested a concept for future research on the relationship between EQ, SQ and peer problems in the community, along with academic difficulties that are usually experienced by children with ADHD. Moreover, this study may add to the understanding of empathy and systemizing abilities in children with ADHD. Therefore, a more comprehensive therapy to target these abilities in clinical practice is needed with regard to this population, rather than targeting only the symptoms of inattention, hyperactivity, and/or impulsive behavior.

## Figures and Tables

**Table 1:** Characteristics of research subjects (parents).

Variable	With ADHD Group (*n* = 61)		Without ADHD Group (*n* = 61)		*p*-Value
	Frequency	%	Frequency	%	
Boys					
Father’s Education Level					
Elementary school	3	6.5	0	0.0	
Junior high school	4	8.7	1	3.2	
Senior high school	19	41.3	24	77.4	0.231
Associate Degree	6	13.0	2	6.5	
Bachelor or more	12	26.1	4	12.9	
No Answer	2	4.3	0	0.0	
Mother’s Education Level					
Elementary school	2	4.3	0	0.0	
Junior high school	5	10.9	3	9.7	
Senior high school	20	43.5	18	58.1	
Associate Degree	5	10.9	5	16.1	0.843
Bachelor or more	13	28.3	5	16.1	
No answer	1	2.2	0	0.0	
Socio-economic status					
Low	12	26.1	7	22.6	
Middle-low	24	52.2	16	51.6	
Middle-High	10	21.7	6	19.4	1.000
High	0	0.0	2	6.5	
Girls					
Father’s Education Level					
Elementary school	1	6.7	0	0.0	
Junior high school	0	0.0	1	3.3	
Senior high school	8	53.3	18	60.0	
Associate Degree	2	13.3	2	6.7	
Bachelor or more	4	26.7	9	30.0	1.000
Mother’s Education Level					
Elementary school	2	13.3	0	0.0	
Junior high school	2	13.3	3	10.0	
Senior high school	6	40.0	17	56.7	
Associate Degree	2	13.3	3	10.0	0.944
Bachelor or more	3	20.0	7	23.3	
Socio-economic status					
Low	5	33.3	3	10.0	
Middle-low	5	33.3	19	63.3	
Middle-High	4	26.7	7	23.3	0.648
High	1	6.7	1	3.3	

**Table 2:** Characteristics of research subjects (children).

Variable	With ADHD Group (*n* = 61)		Without ADHD Group (*n* = 61)		*p*-Value
	Frequency	%	Frequency	%	
Boys					
Children’ age					
7 years old	2	4.3	6	19.4	
8 years old	10	21.7	3	9.7	
9 years old	7	15.2	5	16.1	0.408
10 years old	13	28.3	10	32.3	
11 years old	7	15.2	3	9.7	
≥12 years old	7	15.2	4	12.9	
Grade					
Grade 1–3	28	60.9	15	48.4	0.279
Grade 4–6	18	39.1	16	51.7	
Girls					
Children’ age					
7 years old	1	6.7	9	30.0	
8 years old	2	13.3	4	13.3	
9 years old	7	46.7	5	16.7	0.263
10 years old	1	6.7	8	26.7	
11 years old	1	6.7	1	3.3	
≥12 years old	3	20.0	3	10.0	
Grade					
Grade 1–3	9	60%	15	50%	0.526
Grade 4–6	6	40%	15	50%	

**Table 3:** EQ and SQ score in children with and without ADHD.

	With ADHD Group	Without ADHD Group	Effect Size/ *p*-Value
Boys			
EQ			
Mean (SD)	14.35 (5.88)	17.03 (4.01)	−0.67/0.010 *
Median	13.5	17	
Range	6–33	10–27	
SQ			
Mean (SD)	13.91 (5.4)	15.32 (4.78)	−0.29/0.288 **
Median	14	15	
Range	4–29	9–29	
Girls			
EQ			
Mean (SD)	12.8 (7.36)	18.6 (4.49)	−1.29/0.002 **
Median	13	17	
Range	4–28	12–30	
SQ			
Mean (SD)	10.6 (6.23)	15.27 (4.75)	−0.98/0.008 **
Median	9	14.5	
Range	2–22	9–33	

* Mann-Whitney U test. ** Independent *T*-Test.

**Table 4:** Brain types according to EQ-SQ.

	Brain Type				
	Extreme Empathy	Empathy	Balance	Systemizing	Extreme Systemizing
	**Percentile D < 2.5**	**2.5 ≤ Percentile < 35**	**35 ≤ Percentile < 65**	**65 ≤ Percentile < 97.5**	**Percentile ≥ 97.5**
	**D< −0.16**	**−0.16 ≤ D < −0.016**	**−0.016 ≤ D < 0.029**	**0.029 ≤ D < 0.105**	**D ≥ 0.105**
**Children with ADHD (*n* = 61)**	0	17	13	24	7
**Children without ADHD (*n* = 61)**	1	23	16	20	1

## Data Availability

The data presented in this study are available on request from the corresponding author. The data are not publicly available due to [ethical consideration].
